# Effect of Different Drying Methods on the Quality and Nonvolatile Flavor Components of *Oudemansiella raphanipes*

**DOI:** 10.3390/foods12030676

**Published:** 2023-02-03

**Authors:** Qiulian Shen, Zedong He, Yangyue Ding, Liping Sun

**Affiliations:** Faculty of Food Science and Engineering, Kunming University of Science and Technology, No. 727 South Jingming Road, Kunming 650500, China

**Keywords:** mushroom, drying methods, nonvolatile flavor components, equivalent umami concentration

## Abstract

Different drying methods affect the quality of foods. The aim of this study is to explore the effects of seven drying methods, including hot air drying at 60 °C and 80 °C, ultrasound-assisted hot air drying at 60 °C and 80 °C, microwave drying, vacuum microwave drying, and vacuum freeze-drying, on the quality and nonvolatile flavor components of *Oudemansiella raphanipes*. The vacuum freeze-drying resulted in minimal collapse, mild shrinkage at the macroscopic level, and the formation of uniform pores at the microscopic level on the surfaces of *O. raphanipes* mushrooms. In addition, vacuum freeze-drying can improve the color attributes of the mushrooms. Therefore, the appearance and shape of vacuum freeze-drying treated *O. raphanipes* were closest to those of fresh mushrooms. We found that ultrasound-assisted treatment can effectively shorten the drying time of *O. raphanipes.* The drying time of ultrasound-assisted hot air drying at 60 °C was 20% shorter than that of hot air drying at 60 °C, and the drying time of ultrasound-assisted hot air drying at 80 °C was 37.5% shorter than that of hot air drying at 80 °C. The analysis of the nonvolatile flavor components showed that the ultrasound-assisted hot air drying at 60 °C of the *O. raphanipes* sample had the highest content of free amino acids (83.78 mg/g) and an equivalent umami concentration value (1491.33 monosodium glutamate/100 g). The vacuum freeze-drying treated *O. raphanipes* had the highest 5′-nucleotide content of 2.44 mg/g. Therefore, vacuum freeze-drying and ultrasound-assisted hot air drying at 60 °C, followed by vacuum microwave drying, might protect the flavor components of *O. raphanipes* to the greatest extent. However, microwave drying, hot air drying at 80 °C, and ultrasound-assisted hot air drying at 80 °C could destroy the flavor components of *O. raphanipes* during drying. The results of this study provided data support for the industrial production of dried *O. raphanipes*.

## 1. Introduction

Drying is an ancient and traditional way of preserving food [1]. It is the process of dehydrating food and can effectively decelerate enzyme activity and prevent microbial growth [2]. Drying is often used to treat various fresh vegetables and fruits, such as apples, strawberries, tomatoes, nectarines, mangoes and seabuckthorn [3,4,5,6]. Many studies have shown that drying can affect the structure and flavor components of different food materials. The flavor components include volatile components and nonvolatile components, which determine the product quality of food materials to a certain extent [7,8,9,10,11]. Mushrooms have a short shelf life and are difficult to store and transport. Therefore, drying is an important technology for prolonging the shelf life of mushrooms and facilitating transportation [12]. Li et al. [13] studied the effects of several drying methods on the taste-active compounds of *Pleurotus eryngii* and found that hot air drying and freeze-drying could maximize the retention of these components. Tian et al. [14] found that vacuum microwave drying can improve the quality of dried whole shiitake mushrooms and effectively retain their flavor components. Hu et al. [15] studied the changes in the nonvolatile flavor components of *Stropharia rugoso-annulata* mushrooms that were subjected to different drying methods. In traditional drying methods, pretreatment is often used to shorten the drying time and improve the product quality [16]. Ultrasonication, osmotic dehydration, electroplasmolysis, microwaving and other methods are used for pretreatment before drying to improve the product quality and drying efficiency and reduce the energy consumption [5,17,18,19]. Ruken et al. [19] found that using the combination of electroplasmolysis and ultrasonication to pretreat mushrooms increased the drying rate by 37.10%.

*Oudemansiella raphanipes* is known as a medicinal and edible mushroom. It can be used as a source of human health food because it is rich in nutrients and has a unique flavor and high protein content [20]. The *O. raphanipes* has been successfully domesticated in recent years. In the next few years, the production of *O. raphanipes* will grow rapidly, bringing economic benefits to the world edible fungus industry. In recent years, *O. raphanipes* has gained popularity due to its bioactive and flavor components [21]. Many studies have demonstrated that *O. raphanipes* has a variety of biological activities, including antioxidative, antitumor, immunoregulatory and hepatoprotective activities [22,23]. In addition, the polysaccharides from *O. raphanipes* are used as natural therapy for various metabolic diseases. Xia et al. [21] found that the equivalent umami concentration (EUC) of fresh *O. raphanipes* is 4.72–23.66 monosodium glutamate (MSG)/100 g. 

However, the effect of drying methods on the quality and flavor components of *O. raphanipes* has not been reported. The purpose of this study is to investigate the changes in the flavor components (including 5′-nucleotides, organic acids, and free amino acids) and product quality (appearance and color changes) during the drying of *O. raphanipes* through different methods. The results of this study are helpful for the further industrial production of dried *O. raphanipes*. It also provides theoretical support for the industrial production of other kinds of mushrooms.

## 2. Materials and Methods

### 2.1. Materials and Reagents

Fresh *O. raphanipes* mushrooms were purchased from the local Mushuihua Market (Kunming, China). The standard products of organic and free amino acids were purchased from Chengdu Durst Biotechnology Co., Ltd. (Chengdu, China). The standard products of 5′-nucleotides were purchased from Shanghai Yuanye Biotechnology Co., Ltd. (Shanghai, China). Acetonitrile for mass spectrometry was provided by Merck Drugs and Biotechnology, Ltd. (Beijing, China).

### 2.2. Drying Methods

The *O. raphanipes* samples for the drying experiment were all from the same batch. Soil on the surface of the samples was washed away with running water. 

#### 2.2.1. Hot Air Drying 

A total of 300 g of fresh *O. raphanipes* was dried by using an electric blast oven (101A-1E, Shanghai Experimental Instrument Factory Co., Ltd., Shanghai, China) at 60 °C and 80 °C. After the *O. raphanipes* mushrooms reached a constant weight, two samples were obtained: samples subjected to hot air drying at 60 °C (HAD60) and those subjected to hot air drying at 80 °C (HAD80). The drying time of the HAD60 samples was approximately 10 h, and that of the HAD80 samples was approximately 8 h. 

#### 2.2.2. Ultrasound-Assisted Hot Air Drying

A total of 300 g of fresh *O. raphanipes* was pretreated with an ultrasonic cell grinder (Ningbo Xinzhi Biotechnology Co., Ltd., Ningbo, China), as follows: 20 g of samples were added to 200 mL of ultrapure water and ultrasonicated for 5 min at 200 W. The ultrasonicated *O. raphanipes* was placed in an electric blast oven at 60 °C and 80 °C and dried to a constant weight. The samples subjected to hot air drying at 60 °C and 80 °C were denoted as UA-HAD60 and UA-HAD80, respectively. The drying time of the UA-HAD60 sample was approximately 8 h, and that of the UA-HAD80 sample was approximately 5 h. 

#### 2.2.3. Microwave Drying

Fresh *O. raphanipes* mushrooms were dried by using the microwave drying equipment developed by our laboratory [24]. A total of 300 g of fresh *O. raphanipes* mushrooms were laid flat on a tray at a drying power of 350 W and drying time of 6.5 h. The dried samples were denoted as MD. 

#### 2.2.4. Vacuum Microwave Drying

Fresh *O. raphanipes* mushrooms were dried by using VMD equipment. The VMD equipment was developed by our laboratory [25]. A total of 300 g of fresh *O. raphanipes* mushrooms were laid flat on a tray at a drying power of 350 W and drying time of 4 h. The dried samples were denoted as VMD.

#### 2.2.5. Vacuum Freeze-Drying

A total of 300 g of fresh *O. raphanipes* mushrooms were lyophilized by using a vacuum freeze-dryer (LGJ-12, Beijing Songyuan Huaxing Technology Development Co., Ltd., Beijing, China). The programmed heating mode was adopted for freeze-drying. The cold trap temperature was increased from −55 °C to 35 °C, and the freeze-drying time was 37 h. The dried sample was denoted as VFD. 

### 2.3. Crude Protein and Soluble Protein Analysis

The crude and soluble proteins in all of the dried samples were determined via the Kjeldahl method, in accordance with the National Standard of China (GB 5009.5-2016).

### 2.4. Macrostructure and Microstructure Analyses

The shrinkage and smoothness of the surfaces of the dried *O. raphanipes* samples were analyzed at the macroscopic level. Fully formed dried *O. raphanipes* samples were selected, laid flat on the same surface, and compared with the fresh samples. 

A section of *O. raphanipes* caps was analyzed at the microscopic level by using a scanning electron microscope (SEM) (Inspect F50, Thermo Fisher Scientific Inc., Waltham, MA, USA). The samples were sputtered with gold under vacuum by using a Sputter Coater 108 auto (Cressington Scientific Instruments, Watford, UK).

### 2.5. Color Measurement Analysis

The color changes in the *O. raphanipes* before and after drying were measured using a CR-400 Chroma Meter (Konica Minolta Inc., Tokyo, Japan). The L value represents the lightness of the object, and 0–100 represents blackness to whiteness. A positive a value indicates redness, and a negative a value indicates greenness. A positive b value positive indicates yellowness, and a negative b value indicates blueness.

### 2.6. Hydrophilic Flavor Component Analysis

A total of 0.5 g of dried sample was transferred into a 15 mL centrifuge tube and added with 12 mL of ultrapure water. The mixture was vortexed for 30 s and subjected to ultrasound-assisted extraction for 5 min. Then, it was boiled in boiling water for 5 min. The mixture was centrifuged after cooling (10,000 rpm, 10 min). After being passed through an aqueous membrane, the supernatant was loaded into a liquid-phase vial. Hydrophilic taste-active components (including 5′-nucleotides and free amino acids) in the supernatant were detected using hydrophilic interaction chromatography-triple quadrupole-tandem mass spectrometry (HILIC-QQQ-MS/MS) (LCMS-8040, Shimadzu Inc., Tokyo, Japan).

The methods reported by Wang et al. [26] were used as a reference. The operating conditions of the HILIC were as follows: Syncronis HILIC column (1.7 μm, 2.1 × 100 mm, Thermo Fisher Scientific Inc., Waltham, MA, USA), instrument injection volume of 1 μL, flow rate of 0.30 mL/min, and column temperature of 35 °C. Mobile phase A was 0.2% (*v*/*v*) formic acid and 5 mM ammonium formate ultrapure water, and mobile phase B was 0.2% (*v*/*v*) formic acid and 5 mM ammonium formate acetonitrile–ultrapure water (90:10, *v*/*v*). The elution procedure was as follows: 0–4 min (95–87% B), 4–5.5 min (87–78% B), 5.5–5.7 min (78–70% B), 5.7–6.2 min (70–60% B), 6.2–8.5 min (60–40% B), and 8.5–9.5 min (40–35% B).

The mass spectrometry conditions were as follows: positive ion scan mode (ESI+); spray voltage of 4.5 kV; scavenging flow rate of 3 L/min; drying airflow speed of 15 L/min; ion transfer tube temperature of 300 °C; heating block temperature of 400 °C; collision gas pressure of 230 kPa; and detection voltage of 2.16 kV.

### 2.7. Organic Acid Analysis

A total of 0.4 g of dried sample was placed in 10 mL centrifuge tubes and added with 8 mL of ultrapure water. The mixture was vortexed for 30 s and subjected to ultrasound-assisted extraction for 5 min, then boiled in boiling water for 5 min. The mixture was centrifuged after cooling (10,000 rpm, 10 min). After being passed through an aqueous membrane, the supernatant was loaded into a liquid-phase vial. The organic acids in the supernatant were detected by using ultra-high performance liquid chromatography-triple quadrupole-tandem mass spectrometry (UPLC–QQQ–MS/MS) (LCMS-8040, Shimadzu Inc., Tokyo, Japan).

The methods of the research of Shi et al. [27] were used as a reference. The operating conditions of the UPLC were as follows: Hypercrb C_18_ column (3 μm, 2.1 × 100 mm, Thermo Fisher Scientific Inc., Waltham, MA, USA), instrument injection volume of 1 μL, flow rate of 0.30 mL/min, and column temperature of 45 °C. Mobile phase A was 0.1% (*v*/*v*) formic acid and 1 mM ammonium formate ultrapure water, and mobile phase B was acetonitrile. The elution procedure was as follows: 0–1 min (5–15% B), 1–3.5 min (15–25% B), 3.5–4.4 min (25–35% B), and 4.5–8 min (35–40% B).

The mass spectrometry conditions were as follows: negative ion scan mode (ESI−); spray voltage of 4.5 kV; scavenging flow rate of 3 L/min; drying airflow speed of 15 L/min; ion transfer tube temperature of 250 °C; heating block temperature of 400 °C; CID pressure of 230 kPa; and detection voltage of 2.08 kV.

### 2.8. Statistical Analysis

The experiments were performed with three repetitions. The data were expressed as means ± standard deviation. The data were analyzed using the SPSS software (version 22.0, SPSS Inc., Chicago, IL, USA). The significance of the data was analyzed through an analysis of variance and the Duncan’s multiple range test. A *p* < 0.05 was considered a significant difference.

## 3. Results and Discussion

### 3.1. Macrostructure and Microstructure of O. raphanipes Mushrooms Subjected to Different Drying Methods

**Figure 1:**
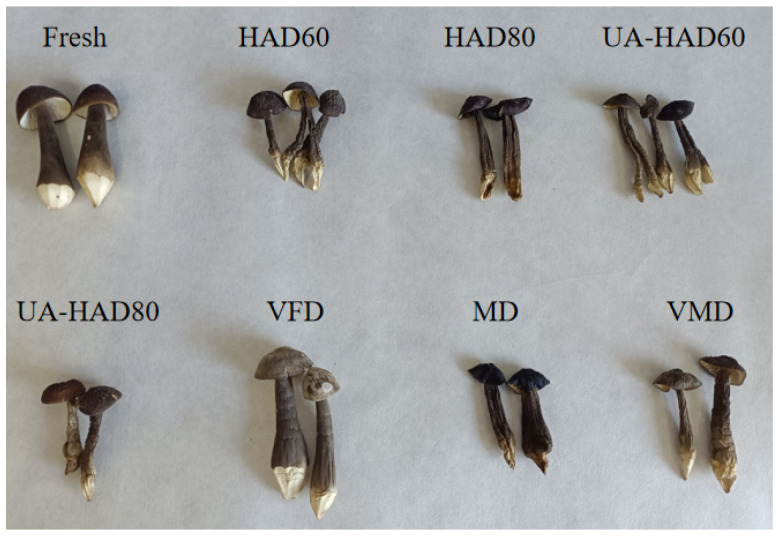
Macroscopic photo of fresh *O. raphanipes* and after drying, HAD60—60 °C hot air drying, HAD80—80 °C hot air drying, UA-HAD60—ultrasonic-assisted 60 °C hot air drying, UA-HAD80—ultrasonic-assisted 80 °C hot air drying, VFD—vacuum freeze drying, MD—microwave drying, VMD—vacuum microwave drying.

Figure 1 shows the macrostructures of the *O. raphanipes* mushrooms processed through seven drying methods compared with those of the fresh samples. The drying methods had a great influence on the shape of the mushrooms. The surfaces of the dried *O. raphanipes* mushrooms exhibited shrinkage. Among the samples, the VFD sample, followed by the VMD sample, showed minimal collapse and mild shrinkage. The surfaces of the MD and HAD80 samples presented severe shrinkage and collapse. Therefore, the shape of the VFD-treated *O. raphanipes* mushrooms was closest to that of fresh mushrooms. 

**Figure 2:**
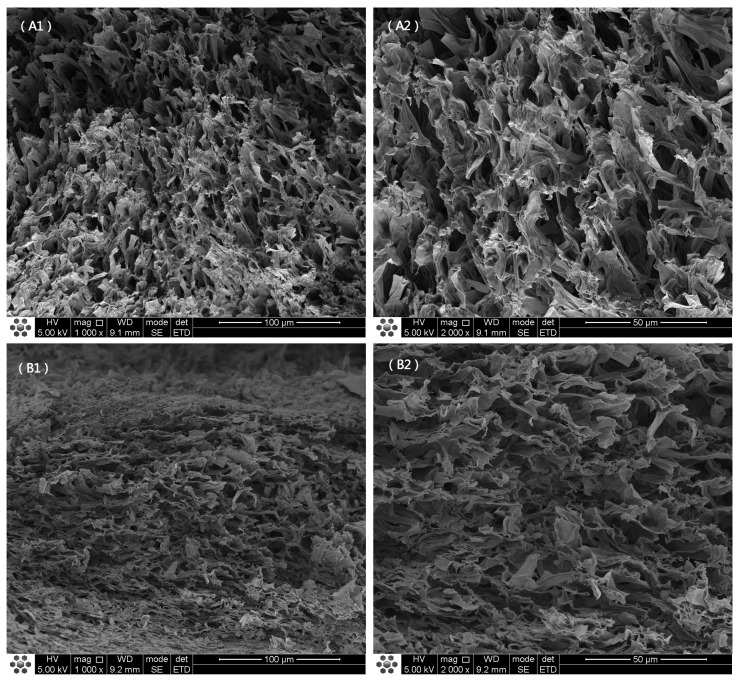

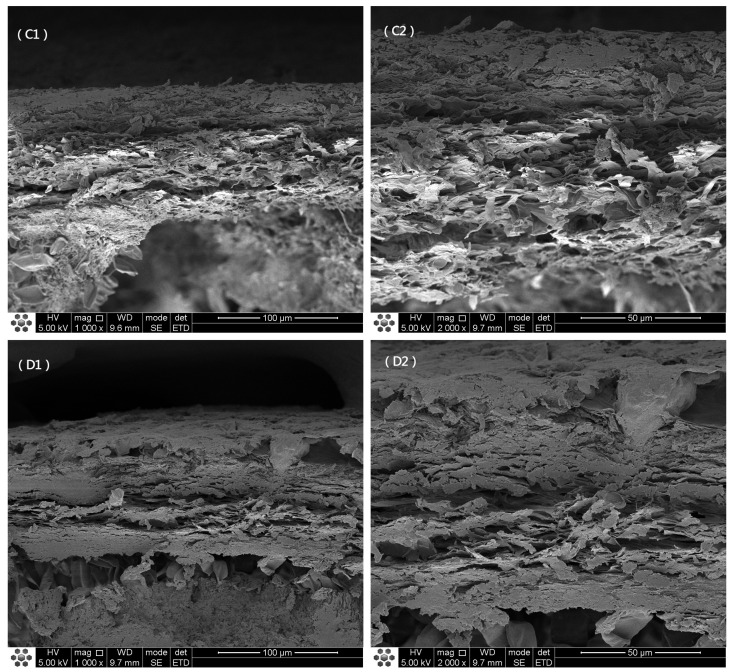

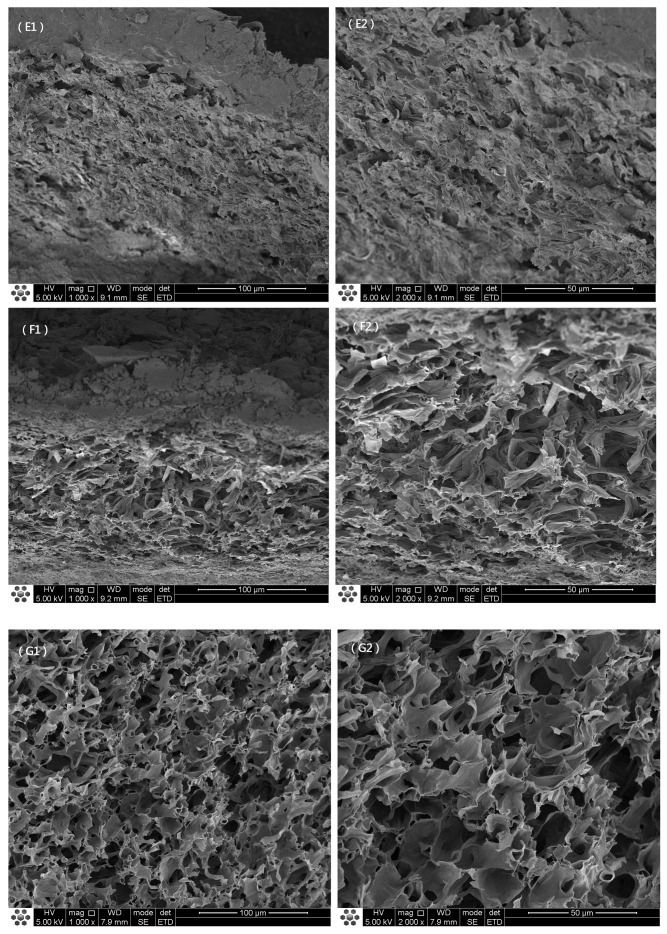
Scanning electron micrographs of different drying methods of *O. raphanipes*. The magnification was set as × 1000 and 2000: (**A1**,**A2**)—ultrasonic-assisted 60 °C hot air drying; (**B1**,**B2**)—60 °C hot air drying; (**C1**,**C2**)—ultrasonic-assisted 80 °C hot air drying; (**D1**,**D2**)—80 °C hot air drying; (**E1**,**E2**)—microwave drying; (**F1**,**F2**)—vacuum microwave drying; (**G1**,**G2**)—vacuum freeze drying.

Figure 2 shows the SEM images of the microstructures of the cap cross-sections of the *O. raphanipes* mushrooms treated through seven drying methods. The results indicated that different drying methods had different influences on the microstructure of the *O. raphanipes.* The dried *O. raphanipes* samples had different microstructures and mainly showed loose pores and flat structures. Figure 2(A1–B2) shows that the microstructure of UA-HAD60 was looser than that of HAD60. The cell wall was speculated to have been broken after ultrasound treatment, resulting in the loose microstructure of UA-HAD60. Similar to this study, previous work, by Zhang et al. [28], found that ultrasound treatment can destroy the cellular structure of mushrooms. Loose pores contributed to the dehydration of the mushrooms. In addition, the cavitation effect produced by the ultrasonication in liquid can effectively remove moisture, which is closely bound to the material. Hence, the drying time of UA-HAD60 was 20% shorter than that of HAD60. Figure 2(C1–D2) illustrate that UA-HAD80 and HAD80 showed collapsed and flattened structures and had harder textures than the other samples. In contrast to UA-HAD60 and HAD60, UA-HAD80 and HAD80 lacked loose microstructures. The results of this study were consistent with those of Zheng et al. [12], who reported that mushrooms presented severe collapse and hardened texture with the increase in temperature. Water was speculated to leave the product rapidly at high temperatures, causing the product to contract sharply. In addition, ultrasound-assisted treatment can effectively shorten the drying time. The drying time of UA-HAD80 was 37.5% shorter than that of HAD80.

Figure 2(E1,E2) show that the microstructure of the MD sample lacked pores. Consistent with the results provided in Figure 1, the MD sample exhibited severe shrinkage and a nonhomogenous structure. Figure 1 illustrates that the surface of the MD sample had undergone severe shrinkage. Li et al. [13] reported that the high-frequency electricity of the microwave increased the central temperature of the samples rapidly and those high temperatures caused the rapid evaporation of the water inside the samples. 

Figure 2(F1,F2) show that the VMD sample exhibited a loose and porous structure in contrast to the MD sample. A vapor pressure difference existed between the mushroom samples and the drying chamber in the vacuum microwave environment [14]. The product was quickly heated by microwave radiation under vapor pressure, resulting in cell expansion and rapid water transfer to the air. These phenomena allowed large interior channels to form in the product. 

Figure 2(G1,G2) show that the VFD sample had more pores with greater uniformity than the other dried samples. The appearance of the VFD sample was similar to that of the fresh mushrooms (Figure 1). The VFD was carried out at a low temperature, and ice crystals were formed in the cells. The high porosity caused by the sublimation of the ice crystals can effectively maintain the integrity of the structure of the mushroom samples. Consistent with our study, the work of Zheng et al. [12] found that the internal pores of freeze-dried *Boletus aereus* samples were significantly larger than those of other dried samples. Pei et al. [29] showed that, in contrast to other drying methods, VFD can reduce the hardness of dried samples because it resulted in the formation of large and uniform internal pores in the samples. 

### 3.2. Protein Content of O. raphanipes Subjected to Different Drying Methods

**Figure 3:**
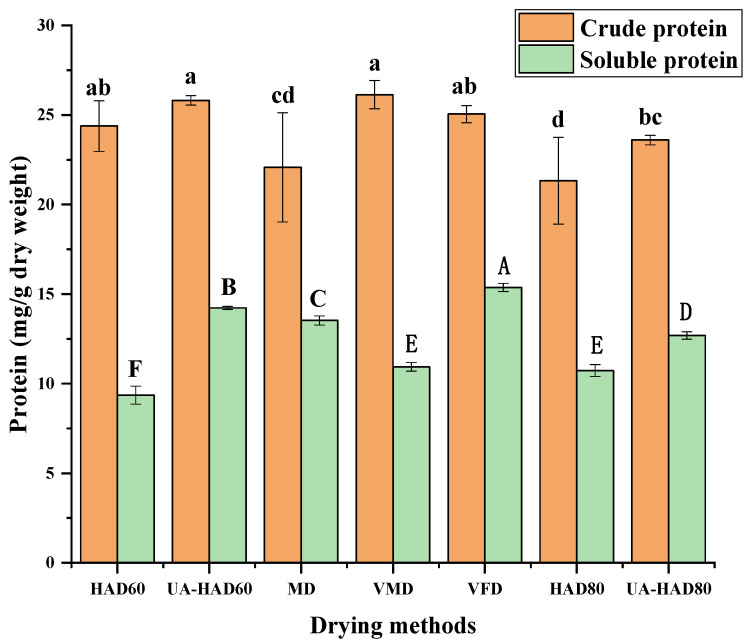
The protein contents of *O. raphanipes* subjected to different drying methods. Different letters in the same line indicate significant difference (*p* < 0.05). Capital letters indicate significance analysis for soluble protein content and small letters indicate significance analysis for crude protein content. HAD60—60 °C hot air drying, HAD80—80 °C hot air drying, UA-HAD60—ultrasonic-assisted 60 °C hot air drying, UA-HAD80—ultrasonic-assisted 80 °C hot air drying, VFD—vacuum freeze drying, MD—microwave drying, VMD—vacuum microwave drying.

Figure 3 shows that the crude protein content of the dried *O. raphanipes* mushrooms ranged between 21.33 mg/g dry weight and 26.13 mg/g dry weight. Previous studies had reported the crude protein contents of commercial mushrooms processed through different drying methods, such as *P. eryngii* (18.21%), *S. rugoso-annulata* (13.19–22.46%) and shiitake mushrooms (1.83–2.69%) [13,14,15]. Their results were similar to our findings. In this work, the VMD sample had the highest crude protein content, of 26.13 mg/g dry weight, whereas the HAD80 sample had the lowest crude protein content, of 21.33 mg/g dry weight. The soluble protein content of the *O. raphanipes* samples treated through different drying methods ranged between 9.36 mg/g dry weight and 15.37 mg/g dry weight. The VFD sample had the highest soluble protein content, and the HAD60 sample had the lowest soluble protein content. These results indicated that the method of drying could have some influence on the protein contents of mushrooms, most likely because heating caused some protein degradation or a Maillard reaction to produce other substances during drying.

### 3.3. Color Measurements of O. raphanipes Treated through Different Drying Methods

Color is one of the standards used to evaluate the quality of dry products, which directly affects consumers’ purchasing desire [30]. Figure 1 shows that the color of the *O. raphanipes* samples treated through different drying methods differed from that of the fresh samples. Among the samples obtained through different drying methods, the MD sample had the highest color intensity and the VFD sample had the lowest color intensity. Table 1 shows the surface color parameters of the *O. raphanipes* samples processed through different drying methods. The L value of the dried *O. raphanipes* mushrooms increased in the order of MD < HAD80 < UA-HAD80 < HAD60 < UA-HAD60 < VMD < VFD. Consistent with the results presented in Figure 1, the VFD sample had the highest L value and the MD sample had the lowest L value among the samples. The low color intensity of the VFD sample may be due to the low oxygen content in a vacuum environment, resulting in reductions in the enzymatic reactions [13]. The methods studied in this work, with the exception of VFD, required a heating process. During heating, nonenzymatic reactions (Maillard reaction) could produce brown substances [31]. This phenomenon was one of the reasons for the darkening of the mushrooms. Furthermore, the color intensity of the HAD80 sample was darker than that of the HAD60 sample, likely because the drying temperature had a certain effect on the color of the mushrooms. This result was consistent with the finding of Guo et al. [32], that is, high temperatures increased the Maillard reaction between sugar and protein, darkening the color of the dried *B. edulis* and reducing the L value. In addition, in the four samples dried by hot air, the a value decreased and the b value increased as the temperature was increased. Therefore, temperature affects the chromaticity and transparency of mushrooms [33].

### 3.4. Free Amino Acids

Free amino acids play an important role in the flavor of mushrooms. The content of free amino acids in the *O. raphanipes* treated through different drying methods are shown in Table 2. Eighteen free amino acids, including six essential amino acids, were detected in the *O. raphanipes* samples subjected to seven different drying methods. The content of total free amino acids in the different samples ranged between 30.80 mg/g and 83.78 mg/g. The content of total free amino acids in the *O. raphanipes* samples increased in the order of UA-HAD80 < MD < HAD80 < VFD < HAD60 < VMD < UA-HAD60. Among the samples, the UA-HAD60 sample had the maximum content of total free amino acids. In addition, the content of free amino acids in the UA-HAD60 sample was higher than that in the HAD60 sample, likely because the ultrasound-assisted treatment facilitated the release of free amino acids. However, the UA-HAD80 sample had the lowest content of free amino acids among the seven dried samples, likely because the carbonyl amine reaction (Maillard reaction) occurred violently at high temperatures, resulting in the serious loss of free amino acids. This result was consistent with the findings of Zheng et al. [12], who discovered that the content of free amino acids in *B. aereus* samples dried by hot air at 60 °C was significantly higher than that in samples dried by hot air at 80 °C. In addition, brief exposure to excessively high temperatures leads to the serious loss of free amino acids in *O. raphanipes* samples subjected to MD.

In accordance with their flavor characteristics, free amino acids are divided into umami amino acids (Glu and Asp), sweet amino acids (Thr, Ser, Pro, Gly, and Ala), bitter amino acids (Val, Ile, Leu, His, Phe, Arg, and Met), and tasteless amino acids (Lys and Tyr). Umami amino acids are the most typical amino acids in mushrooms and are known to have a MSG-like taste. The content of MSG-like umami amino acids in the *O. raphanipes* samples ranged between 13.44 mg/g and 37.68 mg/g. Among the dried *O. raphanipes* samples, the HAD60 sample had the highest umami amino acid contents, whereas the UA-HAD60 sample had the highest content of sweet and bitter amino acids. These results were consistent with those reported by Zheng et al. [12], who found that hot air drying at 60 °C *B. aereus* samples had the highest content of umami amino acids, whereas ultrasound pretreatment combined with hot air drying at 60 °C *B. aereus* samples had the highest content of sweet amino acids. Therefore, HAD60, as a potential drying method, can improve the flavor umami of *O. raphanipes*. In addition, Yang et al. [34] divided MSG-like amino acids into three grades: low (<5 mg/g), medium (5–20 mg/g), and high (>20 mg/g). In this study, four dried samples (HAD60, UA-HAD60, VMD, and MD) had high contents of MSG-like amino acids, whereas the other three dried samples had medium contents of MSG-like amino acids. 

### 3.5. Organic Acids

The organic acids in mushrooms play an important role in flavor. Table 3 shows that a total of ten organic acids were detected, including quinic acid, succinic anhydride, succinic acid, citric acid monohydrate, fumaric acid, maleic acid, citric acid, pyruvic acid, tartaric acid, and lactic acid. The content of total organic acids ranged between 0.73 mg/g and 42.97 mg/g and was higher than that reported for some of the mushrooms reported by Valentao et al. [35], but lower than that of *P. eryngii* reported by Li et al. [13]. Previous studies have shown that differences between mushrooms varieties, as well as different extraction methods and detection methods, lead to differences in the organic acid content [1].

Table 3 shows the significant differences in the organic acid content among the seven dried mushrooms (*p* < 0.05). The contents of the total organic acids of *O. raphanipes* increased in the order of MD < UA-HAD80 < UA-HAD60 < HAD80 < VMD < VFD < HAD60. The content of organic acids in the *O. raphanipes* samples dried through MD and UA-HAD80 were low. Previous studies have demonstrated that high temperature is unconducive to the preservation of organic acids and that decarboxylation occurs during heating, resulting in the loss of organic acids [36]. The use of ultrapure water as the medium in ultrasound-assisted treatment causes the dissolution of hydrophilic organic acids during treatment. Therefore, the total amount of organic acids in the dried mushroom samples subjected to ultrasound-assisted treatment was generally lower than that of the mushroom samples without ultrasound-assisted treatment. The results showed that as a potential drying method, HAD60 can protect the organic acids in *O. raphanipes*. In addition, previous work has shown that the succinic acid is an important flavor substance in food. Succinic acid was detected in all seven mushroom samples in this study. Among them, the VMD sample, followed by the VFD and HAD60 samples, had the highest content of succinic acid.

### 3.6. 5′-Nucleotides

Table 4 shows that five 5′-nucleotides were detected: 5′-AMP, 5′-GMP, 5′-IMP, 5′-XMP, and 5′-CMP. The content of the total 5′-nucleotides of the *O. raphanipes* treated through different drying methods ranged between 0.81 mg/g and 2.44 mg/g. The contents of the total 5′-nucleotides of the *O. raphanipes* increased in the order of HAD80 < UA-HAD80 < MD < HAD60 < UA-HAD60 < VMD < VFD. A previous study indicated that 5′-nucleotides were sensitive to temperature [15]. Most drying methods, except for VFD, need to be carried out at certain temperatures. In the VFD, the low temperature and high porosity caused by ice sublimation prevented the degradation of the 5′-nucleotides in the product [13]. Therefore, the VFD sample had the highest 5′-nucleotide content among the mushroom samples dried through seven different methods. Meanwhile, the 5′-nucleotides in *O. raphanipes* could be degraded by high temperatures or a long heating process, resulting in certain losses. This situation can account for the low 5′- nucleotide contents in the HAD80 and MD samples.

The flavor 5′-nucleotides are mainly 5′-GMP, 5′-IMP, and 5′-XMP [37]. The samples were divided into three grades in accordance with their contents of flavor 5′-nucleotides: low (<1 mg/g), medium (1–5 mg/g), and high (>5 mg/g) [34]. The content of total flavor nucleotides in the dried *O. raphanipes* ranged between 0.50 mg/g and 1.19 mg/g. Therefore, the flavor 5′-nucleotide contents of the UA-HAD60, VMD, and VFD samples were at a medium level, and those of the other four samples were at a low level. 

### 3.7. EUC Value

**Figure 4:**
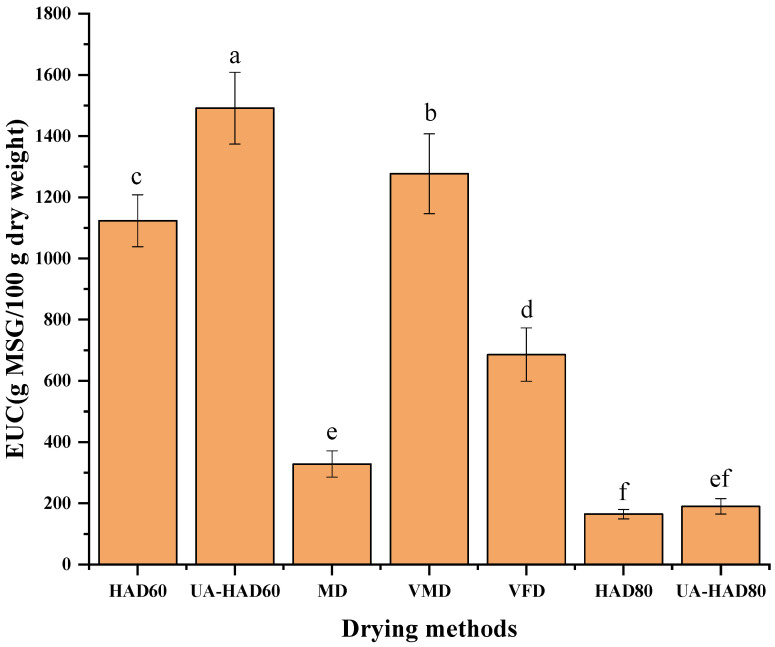
The changes of equivalent umami concentration (EUC) of *O. raphanipes* subjected to different drying methods. Different letters indicate significant difference (*p* < 0.05); Same letter means no significant difference (*p* > 0.05). HAD60—60 °C hot air drying, HAD80—80 °C hot air drying, UA-HAD60—ultrasonic-assisted 60 °C hot air drying, UA-HAD80—ultrasonic-assisted 80 °C hot air drying, VFD—vacuum freeze drying, MWD—microwave drying, VMD—vacuum microwave drying.

The synergistic effect of flavor 5′-nucleotides and umami amino acids greatly improves the umami taste of mushrooms. EUC is quantified by umami amino acids and flavor 5′-nucleotides, among which glutamic acid and 5′-GMP play a major role. Figure 4 shows the EUC value of the *O. raphanipes* samples dried through seven methods. The EUC value of the dried *O. raphanipes* ranged between 164.39 MSG/100 g and 1491.33 MSG/100 g. The EUCs of the dried *O. raphanipes* increased in the order of HAD80 < UA-HAD80 < MD < VFD < HAD60 < VMD < UA-HAD60. The EUC value of the dried *O. raphanipes* treated with hot air at 60 °C was higher than that of the samples treated with hot air at 80 °C. Similarly, the EUC value of the VMD sample was higher than that of the VFD and MD samples. Hence, appropriate heating temperatures contribute to the release of the main EUC-contributing flavor components that can be destroyed by high temperatures. In addition, the EUC value of the samples after ultrasound-assisted treatment was higher than that of the samples that were not subjected to ultrasound-assisted treatment. Ultrasound-assisted treatment shortens the heating time and reduces the destruction of the flavor components by temperature. In addition, it helps release flavor components. Therefore, these results were consistent with the above research results.

## 4. Conclusions

The purpose of this study is to investigate the changes in the flavor components and product quality of *O. raphanipes* during drying through different methods, including HAD80, UA-HAD80, UA-HAD60, HAD60, MD, VFD, and VMD. The different drying methods had significant effects on the flavor component contents and product quality of the mushrooms. Heating can cause the loss of flavor components. The loss of flavor components was particularly severe at elevated temperatures. Ultrasound-assisted treatment shortened the heating time and reduced the destruction of the flavor components. In addition, ultrasound-assisted treatment could help release the flavor components. In the VFD and VMD drying methods, the vapor pressure difference caused the rapid dehydration of the product and allowed the product to develop a porous microstructure. Thus, VMD had the shortest drying time among the seven drying methods. However, VFD required a long drying time and therefore consumed more energy than the other methods. The high-frequency electricity of microwaves resulted in the rapid production of high temperatures in the product. This behavior destroyed the flavor components in *O. raphanipes*. Hence, given their efficient drying rates, low energy consumption, and high EUC value, UA-HAD60 and VMD are potential methods for drying *O. raphanipes*.

## Figures and Tables

**Table 1:** The surface color of *O. raphanipes* subjected to different drying methods.

	UA-HAD60	UA-HAD80	MD	VMD	HAD60	HAD80	VFD
L	47.92 ± 1.50 ^c^	45.04 ± 0.78 ^d^	30.55 ± 0.98 ^f^	62.04 ± 1.54 ^b^	47.71 ± 1.34 ^c^	39.45 ± 1.09 ^e^	67.09 ± 1.07 ^a^
a	0.74 ± 0.52 ^b^	0.51 ± 0.56 ^b^	−4.40 ± 0.91 ^e^	−2.56 ± 0.25 ^c^	2.97 ± 0.62 ^a^	2.47 ± 0.48 ^a^	−4.49 ± 0.52 ^e^
b	16.65 ± 0.22 ^b^	17.91 ± 0.36 ^a^	17.31 ± 1.03 ^ab^	17.73 ± 0.55 ^a^	16.70 ± 0.30 ^b^	17.70 ± 0.44 ^a^	15.61 ± 0.28 ^c^

Data are shown as mean ± SD (*n* = 9). Different letters in the same row indicate significant difference (*p* < 0.05); Same letter means no significant difference (*p* > 0.05). HAD60—60 °C hot air drying, HAD80—80 °C hot air drying, UA-HAD60—ultrasonic-assisted 60 °C hot air drying, UA-HAD80—ultrasonic-assisted 80 °C hot air drying, VFD—vacuum freeze drying, MWD—microwave drying, VMD—vacuum microwave drying. L—lightness coefficient; a—red color coefficient; b—yellow color coefficient.

**Table 2:** The content of free amino acids of *O. raphanipes* subjected to different drying methods (mg/g dry weight).

	HAD60	UA-HAD60	HAD80	UA-HAD80	MD	VMD	VFD
Ala	2.05 ± 0.04 ^c^	2.39 ± 0.12 ^b^	1.38 ± 0.20 ^d^	1.18 ± 0.11 ^e^	1.04 ± 0.03 ^e^	2.83 ± 0.07 ^a^	1.03 ± 0.11 ^e^
Arg	3.46 ± 0.07 ^c^	5.13 ± 0.03 ^a^	1.83 ± 0.06 ^e^	1.23 ± 0.18 ^f^	2.46 ± 0.13 ^d^	4.06 ± 0.09 ^b^	4.13 ± 0.10 ^b^
Glu	36.88 ± 2.44 ^a^	32.34 ± 2.66 ^b^	17.80 ± 0.51 ^d^	12.78 ± 1.86 ^e^	20.40 ± 0.78 ^d^	26.99 ± 2.06 ^c^	13.64 ± 1.14 ^e^
Asp	0.80 ± 0.03 ^d^	0.70 ± 0.01 ^e^	1.09 ± 0.06 ^b^	0.65 ± 0.04 ^e^	1.01 ± 0.09 ^c^	0.62 ± 0.04 ^e^	1.35 ± 0.04 ^a^
His *	1.82 ± 0.05 ^c^	2.76 ± 0.03 ^a^	1.11 ± 0.02 ^d^	1.19 ± 0.11 ^d^	1.23 ± 0.10 ^d^	2.11 ± 0.02 ^b^	1.85 ± 0.08 ^c^
Val *	2.57 ± 0.12 ^a^	2.80 ± 0.04 ^a^	1.86 ± 0.17 ^b^	1.44 ± 0.42 ^c^	1.51 ± 0.05 ^c^	2.12 ± 0.04 ^b^	1.51 ± 0.09 ^c^
Thr *	0.73 ± 0.02 ^b^	0.86 ± 0.03 ^a^	0.07 ± 0.01 ^e^	0.08 ± 0.01 ^e^	0.12 ± 0.01 ^e^	0.67 ± 0.04 ^c^	0.53 ± 0.05 ^d^
Tyr	1.18 ± 0.06 ^c^	1.68 ± 0.09 ^b^	0.84 ± 0.06 ^d^	0.79 ± 0.25 ^de^	0.60 ± 0.02 ^e^	2.31 ± 0.06 ^a^	2.13 ± 0.11 ^a^
Hser	1.36 ± 0.03 ^b^	1.72 ± 0.07 ^a^	1.04 ± 0.03 ^c^	0.89 ± 0.11 ^de^	0.84 ± 0.03 ^e^	1.32 ± 0.07 ^b^	0.98 ± 0.12 ^cd^
GABA	1.41 ± 0.01 ^b^	1.64 ± 0.15 ^a^	0.21 ± 0.00 ^de^	0.31 ± 0.03 ^d^	0.21 ± 0.02 ^de^	1.10 ± 0.08 ^c^	0.15 ± 0.02 ^e^
Pro	1.60 ± 0.11 ^a^	1.14 ± 0.02 ^b^	1.40 ± 0.04 ^a^	0.83 ± 0.29 ^c^	0.72 ± 0.04 ^c^	1.10 ± 0.05 ^b^	0.88 ± 0.02 ^c^
Ile *	1.87 ± 0.04 ^ab^	1.87 ± 0.01 ^ab^	2.29 ± 0.09 ^a^	1.18 ± 0.85 ^c^	0.80 ± 0.09 ^c^	1.37 ± 0.05 ^bc^	1.06 ± 0.08 ^c^
Ser	1.49 ± 0.04 ^b^	1.71 ± 0.06 ^a^	1.37 ± 0.06 ^c^	1.44 ± 0.06 ^bc^	1.49 ± 0.03 ^b^	1.40 ± 0.03 ^bc^	1.04 ± 0.07 ^d^
Leu *	3.52 ± 0.11 ^a^	3.49 ± 0.03 ^a^	2.34 ± 0.05 ^b^	1.31 ± 0.72 ^c^	1.12 ± 0.06 ^c^	3.91 ± 0.21 ^a^	3.66 ± 0.34 ^a^
Lys *	1.43 ± 0.05 ^e^	12.81 ± 0.08 ^a^	1.14 ± 0.02 ^e^	3.79 ± 0.19 ^d^	1.21 ± 0.09 ^e^	8.62 ± 0.43 ^c^	10.11 ± 0.38 ^b^
Gly	0.38 ± 0.01 ^a^	0.31 ± 0.02 ^b^	0.34 ± 0.01 ^b^	0.32 ± 0.02 ^b^	0.41 ± 0.01 ^a^	0.19 ± 0.02 ^c^	0.14 ± 0.03 ^d^
Asn	1.51 ± 0.02 ^c^	1.63 ± 0.03 ^b^	1.01 ± 0.03 ^e^	0.87 ± 0.09 ^f^	0.96 ± 0.07 ^e^	1.78 ± 0.02 ^a^	1.31 ± 0.05 ^d^
Gln	0.91 ± 0.04 ^d^	8.81 ± 0.11 ^a^	0.49 ± 0.06 ^e^	0.52 ± 0.14 ^e^	0.23 ± 0.06 ^e^	5.72 ± 0.25 ^c^	6.58 ± 0.37 ^b^
Umami	37.68 ± 2.46 ^a^	33.04 ± 2.67 ^b^	18.89 ± 0.56 ^d^	13.44 ± 1.89 ^e^	21.41 ± 0.87 ^d^	27.61 ± 2.06 ^c^	14.98 ± 1.15 ^e^
Sweetness	6.24 ± 0.08 ^a^	6.40 ± 0.12 ^a^	4.56 ± 0.25 ^b^	3.86 ± 0.43 ^c^	3.78 ± 0.10 ^c^	6.20 ± 0.02 ^a^	3.62 ± 0.25 ^c^
Bitterness	13.24 ± 0.07 ^b^	16.05 ± 0.03 ^a^	9.43 ± 0.24 ^c^	6.34 ± 1.91 ^d^	7.12 ± 0.35 ^d^	13.57 ± 0.29 ^b^	12.21 ± 0.62 ^b^
Total	64.98 ± 2.48 ^b^	83.78 ± 2.27 ^a^	37.62 ± 0.62 ^d^	30.80 ± 2.51 ^e^	36.36 ± 1.46 ^d^	68.22 ± 2.99 ^b^	52.08 ± 2.64 ^c^

Data are shown as mean ± SD (*n* = 3). Different letters in the same row indicate significant difference (*p* < 0.05); Same letter means no significant difference (*p* > 0.05). HAD60—60 °C hot air drying, HAD80—80 °C hot air drying, UA-HAD60—ultrasonic-assisted 60 °C hot air drying, UA-HAD80—ultrasonic-assisted 80 °C hot air drying, VFD—vacuum freeze drying, MWD—microwave drying, VMD—vacuum microwave drying. Ala—Alanine, Arg—Argnine, Glu—Glutamic acid, Asp—Aspartic acid, His—Histidine, Val—Valine, Thr—L-Threonine, Tyr—Tyrosine, Hser—L-Homoserine, GABA—γ-aminobutyric acid, Pro—Proline, Ile—L-isoleucine, Ser—Serine, Leu—Leucine, Lys—Lysine, Gly—Glycine, Asn—Asparagine, Gln—Glutamine. * Essential amino acids. Umami: Glu + Asp; Sweetness: Thr + Ser + Pro + Gly + Ala; Bitterness: Val + Ile + Leu + His + Arg.

**Table 3:** The content of organic acids of *O. raphanipes* subjected to different drying methods (mg/g dry weight).

	HAD60	UA-HAD60	HAD80	UA-HAD80	MD	VMD	VFD
Quinic acid	10.03 ± 0.01 ^a^	0.42 ± 0.01 ^e^	1.11 ± 0.01 ^b^	0.05 ± 0.01 ^f^	0.03 ± 0.00 ^f^	0.75 ± 0.03 ^d^	0.96 ± 0.09 ^c^
Succinic anhydride	4.73 ± 0.05 ^a^	0.53 ± 0.06 ^d^	1.09 ± 0.04 ^c^	0.14 ± 0.00 ^e^	0.05 ± 0.01 ^e^	4.49 ± 0.10 ^b^	4.67 ± 0.07 ^a^
Succinic acid	3.87 ± 0.02 ^b^	0.37 ± 0.02 ^cd^	0.69 ± 0.02 ^c^	0.09 ± 0.00 ^d^	0.05 ± 0.01 ^d^	4.37 ± 0.14 ^a^	3.98 ± 0.48 ^b^
Citric acid monohydrate	3.78 ± 0.04 ^a^	0.20 ± 0.01 ^e^	0.38 ± 0.01 ^c^	0.16 ± 0.00 ^e^	0.16 ± 0.00 ^e^	0.30 ± 0.00 ^d^	0.43 ± 0.06 ^b^
Fumaric acid	0.75 ± 0.00 ^d^	0.87 ± 0.02 ^b^	1.79 ± 0.01 ^a^	0.23 ± 0.03 ^f^	nd	0.81 ± 0.02 ^c^	0.68 ± 0.01 ^e^
Maleic acid	0.91 ± 0.00 ^d^	1.67 ± 0.02 ^a^	1.19 ± 0.04 ^b^	0.38 ± 0.03 ^f^	0.22 ± 0.03 ^g^	1.02 ± 0.06 ^c^	0.83 ± 0.03 ^e^
Citric acid	3.01 ± 0.19 ^a^	1.62 ± 0.02 ^c^	2.11 ± 0.05 ^b^	0.42 ± 0.02 ^e^	nd	0.97 ± 0.01 ^d^	1.56 ± 0.18 ^c^
Pyruvic acid	3.61 ± 0.01 ^a^	0.44 ± 0.00 ^d^	0.75 ± 0.00 ^b^	nd	nd	nd	0.67 ± 0.02 ^c^
Tartaric acid	4.61 ± 0.01 ^a^	0.28 ± 0.01 ^c^	0.64 ± 0.01 ^b^	0.10 ± 0.00 ^e^	0.08 ± 0.00 ^f^	0.10 ± 0.00 ^e^	0.11 ± 0.00 ^d^
Lactic acid	7.67 ± 0.41 ^a^	0.63 ± 0.01 ^c^	1.39 ± 0.01 ^b^	0.20 ± 0.01 ^d^	0.14 ± 0.02 ^d^	0.13 ± 0.02 ^d^	0.10 ± 0.01 ^d^
Total	42.97 ± 0.42 ^a^	7.01 ± 0.04 ^e^	11.14 ± 0.07 ^d^	1.76 ± 0.03 ^f^	0.72 ± 0.05 ^g^	12.93 ± 0.14 ^c^	13.97 ± 0.24 ^b^

Data are shown as mean ± SD (*n* = 3). Different letters in the same row indicate significant difference (*p* < 0.05); Same letter means no significant difference (*p* > 0.05). HAD60—60 °C hot air drying, HAD80—80 °C hot air drying, UA-HAD60—ultrasonic-assisted 60 °C hot air drying, UA-HAD80—ultrasonic-assisted 80 °C hot air drying, VFD—vacuum freeze drying, MWD—microwave drying, VMD—vacuum microwave drying. nd = not detected.

**Table 4:** The content of 5′-nucleotide of *O. raphanipes* subjected to different drying methods (mg/g dry weight).

	HAD60	UA-HAD60	HAD80	UA-HAD80	MD	VMD	VFD
5′-AMP	0.42 ± 0.02 ^c^	0.65 ± 0.03 ^b^	0.19 ± 0.00 ^e^	0.30 ± 0.03 ^d^	0.33 ± 0.04 ^d^	0.62 ± 0.03 ^b^	1.16 ± 0.02 ^a^
5′-GMP	0.38 ± 0.01 ^c^	0.56 ± 0.03 ^ab^	0.24 ± 0.02 ^d^	0.40 ± 0.03 ^c^	0.43 ± 0.05 ^c^	0.59 ± 0.03 ^a^	0.50 ± 0.04 ^b^
5′-CMP	0.17 ± 0.01 ^b^	0.26 ± 0.02 ^a^	0.12 ± 0.02 ^c^	0.09 ± 0.01 ^d^	0.09 ± 0.00 ^d^	0.24 ± 0.02 ^a^	0.24 ± 0.01 ^a^
5′-IMP	0.02 ± 0.00 ^d^	0.04 ± 0.01 ^b^	0.01 ± 0.00 ^e^	0.02 ± 0.00 ^d^	0.02 ± 0.01 ^d^	0.03 ± 0.00 ^c^	0.07 ± 0.01 ^a^
5′-XMP	0.35 ± 0.02 ^d^	0.54 ± 0.03 ^a^	0.25 ± 0.03 ^e^	0.37 ± 0.03 ^cd^	0.41 ± 0.04 ^bc^	0.57 ± 0.03 ^a^	0.46 ± 0.06 ^b^
Flavor 5′-nucleotide	0.75 ± 0.03 ^d^	1.13 ± 0.04 ^a^	0.50 ± 0.06 ^e^	0.79 ± 0.06 ^cd^	0.86 ± 0.05 ^c^	1.19 ± 0.06 ^a^	1.03 ± 0.09 ^b^
Total	1.33 ± 0.06 ^c^	2.04 ± 0.08 ^b^	0.81 ± 0.07 ^d^	1.18 ± 0.09 ^c^	1.29 ± 0.09 ^c^	2.05 ± 0.09 ^b^	2.44 ± 0.10 ^a^

Data are shown as mean ± SD (*n* = 3). Different letters in the same row indicate significant difference (*p* < 0.05); Same letter means no significant difference (*p* > 0.05). HAD60—60 °C hot air drying, HAD80—80 °C hot air drying, UA-HAD60—ultrasonic-assisted 60 °C hot air drying, UA-HAD80—ultrasonic-assisted 80 °C hot air drying, VFD—vacuum freeze drying, MWD—microwave drying, VMD—vacuum microwave drying. 5′-AMP—adenosine 5’-monophosphate, 5′-GMP—guanosine 5’-monophosphate, 5′-CMP—cytidine 5′-monophosphate, 5′-IMP—inosine 5’-monophosphate, 5′-XMP—xanthosine 5′-monophosphate. Flavor 5′-nucleotides = 5′-GMP + 5′-IMP + 5′-XMP.

## Data Availability

The data are available from the corresponding author.
